# Predictors of consent to cell line creation and immortalisation in a South African schizophrenia genomics study

**DOI:** 10.1186/s12910-018-0313-2

**Published:** 2018-07-11

**Authors:** Megan M. Campbell, Jantina de Vries, Sibonile G. Mqulwana, Michael M. Mndini, Odwa A. Ntola, Deborah Jonker, Megan Malan, Adele Pretorius, Zukiswa Zingela, Stephanus Van Wyk, Dan J. Stein, Ezra Susser

**Affiliations:** 1 Department of Psychiatry and Mental Health, University of Cape Town, J-Block, Groote Schuur Hospital, Observatory, Cape Town, South Africa; 2 Department of Medicine, University of Cape Town, Groote Schuur Hospital, Observatory, Cape Town, 8000 South Africa; 3 Department of Psychiatry and Behavioural Sciences, Walter Sisulu University, Mthatha, South Africa; 4 Mailman School of Public Health, Columbia University and New York State Psychiatric Institute, New York, USA

**Keywords:** Neuropsychiatric genomics, Xhosa, Consent, Cell immortalisation, Predictors

## Abstract

**Background:**

Cell line immortalisation is a growing component of African genomics research and biobanking. However, little is known about the factors influencing consent to cell line creation and immortalisation in African research settings. We contribute to addressing this gap by exploring three questions in a sample of Xhosa participants recruited for a South African psychiatric genomics study: First, what proportion of participants consented to cell line storage? Second, what were predictors of this consent? Third, what questions were raised by participants during this consent process?

**Methods:**

760 Xhose people with schizophrenia and 760 controls were matched to sex, age, level of education and recruitment region. We used descriptive statistics to determine the proportion of participants who consented to cell line creation and immortalization. Logistic regression methods were used to examine the predictors of consent. Reflections from study recruiters were elicited and discussed to identify key questions raised by participants about consent.

**Results:**

Approximately 40% of participants consented to cell line storage. The recruiter who sought consent was a strong predictor of participant’s consent. Participants recruited from the South African Eastern Cape (as opposed to the Western Cape), and older participants (aged between 40 and 59 years), were more likely to consent; both these groups were more likely to hold traditional Xhosa values. Neither illness (schizophrenia vs control) nor education (primary vs secondary school) were significant predictors of consent. Key questions raised by participants included two broad themes: clarification of what cell immortalisation means, and issues around individual and community benefit.

**Conclusions:**

These findings provide guidance on the proportion of participants likely to consent to cell line immortalisation in genomics research in Africa, and reinforce the important and influential role that study recruiters play during seeking of this consent. Our results reinforce the cultural and contextual factors underpinning consent choices, particularly around sharing and reciprocity. Finally, these results provide support for the growing literature challenging the stigmatizing perception that people with severe mental illness are overly vulnerable as a target group for heath research and specifically genomics studies.

**Electronic supplementary material:**

The online version of this article (10.1186/s12910-018-0313-2) contains supplementary material, which is available to authorized users.

## Background

Cell line creation and immortalisation is a growing component of African genomics research and biobanking. The process involves altering the genetic make-up of a human body cell to allow it to continue reproducing indefinitely, outside of the human body; providing an inexhaustible resource for DNA and other cell products [1]. These products can then be used for a wide range of functional genetics research [2]. Cell line creation and immortalisation in African genomics studies would promote African representation in future functional genomics work, increase the pertinence of discoveries and therapeutic applications to African populations, while reducing the burden and cost to African researchers and participants relating to additional sample donations [1].

While some theoretical work has been done to explore the broader ethical considerations relating to such practice [1, 3], there is little empirical work describing the predictors of consent to cell line creation and immortalisation in African research settings. Many research participants from low and middle income countries (LMICs) contend with the challenges of poverty, limited access to healthcare, low research and health literacy levels and unfamiliarity with the research process, all of which may influence the consent process for African genomic research and biobanking [4–6]. One reported challenge for seeking informed consent for health research in LMIC settings is that procedures tend to be complex and understanding of research study elements varies considerably across participants [7, 8] – a challenge that has also been identified in relation to informed consent for African genomics research [5, 6, 9]. An influential power dynamic between clinicians and the participants being recruited for research has also been reported [7, 10]. However we are unsure as to the influence of these factors on choices about consent to the creation of cell lines for long-term sample storage and re-use in the African setting. This article aims to contribute to addressing this gap by examining consent for cell immortalisation during enrollment into a South African psychiatric genomics study. This paper is a secondary analysis, using data collected from the Genomics of Schizophrenia in South African Xhosa People (SAX) project. Specifically, we consider the following questions: First, what proportion of participants consented to cell line storage? Second, what were predictors of this consent? Third what questions were raised by participants about this consent during recruitment?

## Methods

### The Genomics of Schizophrenia in South African Xhosa people (SAX) project

As is the case for genomic research in general [11] current psychiatric genomics research is under-representative of African populations [12]. The SAX study aims to identify mutations underlying predisposition to schizophrenia in the Xhosa people. This study is designed to initiate this kind of genomics research in Africa, and moreover, will be informative for genomics research on schizophrenia worldwide [13].

### Recruitment and data collection

The SAX study recruits Xhosa people with schizophrenia and Xhosa controls matched to sex, age, education level and region recruited. Cases are recruited from provincial psychiatric hospitals and clinics in the Eastern and Western Cape provinces of South Africa, and include both in and out-patients. Participants must have fulfilled the diagnostic criteria for schizophrenia or schizoaffective disorder over at least a two-year period. Controls are recruited from university-affiliated general medical hospitals and community health centers that draw from similar catchment areas to the psychiatric hospitals.

All participants complete the University of California, San Diego Brief Assessment of Capacity to Consent Questionnaire (UBACC), a 10-item tool for evaluating quality of understanding of different SAX study elements, and identifying areas where participants require further explanation [14, 15]. Next cases and controls complete the Structured Diagnostic Interview for DSM-IV Axis I Disorders (SCID-I) [16], along with other psychiatric measures. All recruitment and clinical interview materials are administered in Xhosa, having been translated in accordance with the World Health Organization (WHO) translation guidelines [17]. Participants then provide blood samples for DNA analysis and HIV screening.

The SAX study uses a staged consent model where all consenting participants agree to participate in the primary genomic study. This involves a blood sample for DNA analysis in the form of next generation sequencing. Participants also consent at this stage to HIV testing in order to account for any HIV related neurological complications. Next, participants receive information about sample (DNA) storage which they can choose to consent to. As a third step, participants are given information about cell immortalisation. This is an optional component requiring an additional blood sample, and a refusal of cell line creation does not affect eligibility to participate in the primary genomic study. All three stages of consent are discussed in person with the study recruiter at the time of recruitment. Recruiters review the consent information sheets with participants in Xhosa.

The information sheet outlining consent to cell line creation provides the following explanation about cell immortalisation:

***“What does cell immortalisation entail?*** *With your permission, we would like to store your blood cells in a way that they last forever (this is called “immortalisation of cell lines”). This would make it possible for researchers to study the DNA in your blood cells in the future. DNA forms a set of instructions, and in the future scientists could look at these instructions in more detail. Right now, we do not know what these ways of working may be. It is possible that future work would lead to new information and treatments of illnesses.*

***Potential risks and benefits:*** *The reason somebody might want to donate their cells is that it may allow many scientists and researchers to learn about illnesses far into the future. However immortalised cells can last forever. These cells are also a more detailed and accurate copy of your DNA. If you wish to withdraw your data or your sample in the future this is possible. However, please note that by the time we withdraw your data or your sample, it may already have been shared with other scientists.*

***Compensation:*** *You will not receive any further compensation for donating your cells.*

***What will happen with the information you provide:*** *Your immortalised cells and some of your information will be shared with other scientists. Your identifying information will not be shared.”*

### Data analysis

Participants recruited for the SAX study from March 2015 to November 2016 were included for analysis. Descriptive statistics established the proportion of participants who consented to cell line immortalisation. Logistic regression methods were used to examine the predictors of consent to cell line creation and immortalization. Consent to cell line creation and immortalisation was used as the outcome variable. We hypothesized that the recruiter would play a significant role in consent [7]. SAX study recruiters were all first language Xhosa speaking, professional psychiatric nurses with clinical experience in a range of psychiatric disorders. These recruiters ranged in age from 28 to 45 years, including one younger female psychiatric nurse, and four male nurses of varying ages. All recruiters lived in the Western Cape but most had lived in the Eastern Cape for a period or had family living in the province. However, none of the nurses had any previous experience engaging with the psychiatric in- and out-patients at the sites targeted for the SAX study. Anonymous letter codes were assigned to each study recruiter (A-E). The recruiter reporting the lowest consent rates was used as the reference nurse for the regression analysis.

The following additional categorical variables were included in our analysis to explore possible associations: absence/presence of a schizophrenia diagnosis (cases - diagnosis of schizophrenia/ schizoaffective disorder or controls - those presenting for treatment of other health concerns), sex (male or female), age group (20–39 years or 40–59 years), education level (primary school – achieved Grade 7 or less; secondary school – achieved Grade 8 or more), region recruited from (Western Cape or Eastern Cape) and HIV status (non-reactive or reactive). Data analysis was generated and managed using IBM SPSS Statistics. Study recruiter reflections on key questions about cell line creation posed by participants during recruitment were recorded during a team discussion and salient themes highlighted.

## Results

### Proportion and predictors of consent to cell line storage

The SAX study recruited a total of 1520 participants from March 2015 to November 2016 or 760 matched pairs of cases and controls, which were included for our analysis here. Of these, 360 (23,6%) participants consented to DNA storage for the SAX study only, 512 (33,6%) consented to broad sample storage and 648 (42.6%) agreed to the collection of an additional blood sample for cell line creation. This subsample of participants who consented to cell line creation included 308 (40.5%) cases with schizophrenia and 340 (44.7%) unaffected controls. The study recruiter was the strongest predictor of consent to cell line creation and immortalisation. Other predictors of consent included older age (40–59 years) and recruitment from the Eastern Cape. A diagnosis of schizophrenia, sex, education level and HIV status did not predict consent. Results are summarized in Table 1 (Additional file 1).

**Table 1:** Sample information, prevalence ratios and odds ratios of consent to cell line creation

Sample information	Categories	N (%)	Consent to cell lines N (%)	Odds Ratio	CI: 95%
Total sample		1520 (100%)	648 (42.6%)		
Absence/presence of schizophrenia diagnosis	Controls	760 (50%)	340 (44.7%)	1.210	0.959–1.527
	Cases	760 (50%)	308 (40.5%)		
Sex:	Female	184 (12.1%)	84 (45.7%)	1.166	0.822–1.655
	Male	1336 (87.9%)	564 (42.2%)		
Age:	40–59	532 (35%)	252 (47.4%)	1.308*	1.020-1.676
	20–39	988 (65%)	396 (40.1%)		
Education level:	Secondary (≥Grade 8)	1130 (74.3%)	485 (42.9%)	1.041	0.791–1.370
	Primary (≤Grade 7)	390 (25.7%)	163 (41.8%)		
Recruitment region:	Eastern Cape	878 (57.8%)	408 (46.5%)	1.597**	1.257-2.027
	Western Cape	642 (42.2%)	240 (37.4%)		
HIV status	Reactive	185 (12.2%)	102 (55.1%)	1.306	0.912–1.871
	Non-reactive	1335 (87.8%)	546 (40.9%)		
SAX Recruiters					
	A	308 (20.3%)	230 (74.7%)	12.587**	8.815–17.972
	B	437 (28.8%)	229 (52.4%)	4.802**	3.530–6.532
	C	207 (13.6%)	74 (35.7%)	2.567**	1.754–3.757
	D	130 8.6%)	29 (22.3%)	1.199	0.739–1.944
	E (reference nurse)	438 (28.8%)	86 (19.6%)		

*p < 0.05

**p < 0.01

### Questions raised during the consent process

During the course of the SAX genomics project, we held regular quality control and supervision meetings with the study recruiters. At these meetings, we discussed challenges encountered in enrollment, overall experiences, personal opinions and also solicited feedback on the consent process. We asked recruiters to recall questions raised by participants during the consenting process to cell line storage and immortalization. These questions were noted and thematically analysed. Key questions retrospectively recalled by our study recruiters, focused on two broad themes. The first theme pertained to clarification of information already outlined in the information sheet. Such questions included further explanations about the cell line immortalisation process and how it differed from DNA storage; clarification about whether it was compulsory to donate blood samples for cell line storage, where the blood would be stored and for how long. Explanations of aspects of cell line immortazation are complex and these recruiter experiences highlight the importance of an iterative approach during consent where the same recruiter identifies unclear aspects of the study the participant may be struggling with, revisits and clarifies these details using alternative examples and explanations when necessary.

A second theme pertained to benefit i.e.: who would be benefitting from the storage of these samples and in what ways? Examples included: What will the blood be used for? Will I benefit from the future research that may be done using these cells? Why will the samples be stored in the US, and why for an indefinite period of time? Can these samples be used by or sold to other people, and if so how can you be sure these cells will be used for the purposes initially intended? Do you trust the people you are giving these cells to?

## Discussion

Approximately 40% of participants in our study consented to cell line creation and immortalisation, providing future researchers with an indication of expected consent rates. The study recruiter was the strongest predictor of consent to cell line immortalisation. This may be due to personal perspectives recuriters hold about the cell immortalisation process, influencing how risks and benefits are emphasized and reiterated to participants during recruitment.

During discussions about challenges encountered in enrollment, over time, we noted a marked difference between two recruiters in their views on cell immortalization. One reported concern about the long term protection and use of participant samples, while the other was more supportive of the potential scientific advances that could be made as a result of cell line immortalization and how these may eventually benefit the Xhosa community. When we analysed consent rates, we noted that the more critical nurse consented fewer participants to cell immortalization (Recruiter E, *n* = 86, 20%) than the more supportive one (Recruiter A, *n* = 230, 75%). Interestingly, Recruiters B and C, who tended to take a more balanced perspective on both the risks and benefits of cell immortalization maintained a consent rate of 52% (*n* = 229) and 36% (*n* = 74) respectively.

While we did not monitor the consent process to explore exactly how much time each recruiter spent on explaining the various aspects of the procedure, it is likely that the more critical recruiter spent more time describing risks and challenges, whilst the more supportive recruiter spent more time describing potential (scientific) benefits. This finding illustrates the subtle influence recruiters hold over participants, and the need to be aware of and manage this potential bias during recruitment. In retrospect a more thoroughly written explanation of potential risks and benefits in the information sheet may have assisted here. In addition, regular follow-ups on individual recruiter consent rates during quality control meetings would have assisted in addressing reasons for significant differences across recruiters earlier on.

Older age was a second predictor of consent to cell line creation and immortalisation in our sample. Overall 47,4% of participants aged 40–59 years consented to cell line immortalization in comparison with 40,1% of participants aged 20–39 years. One explanation for this may involve the power dynamic reported between the recruiter and the participant, pertaining specifically to the recruiter’s perceived competency [7, 10]. Some recruiters may be perceived as having more knowledge and holding more social status than the participant in particular Xhosa communities. Within these communities there is a strong emphasis on respect for social role. In these settings the recruiter may be perceived by the participant to be acting in their best interest. As a result the participant consents to what they perceive the recruiter to be suggesting. This may be particularly true of older participants who may subscribe more closely to these traditional roles, and in so doing forgo some degree of voluntariness in their consent. A deeper awareness of these cultural norms encourages recruiters to gently address this power dynamic when they recognize it playing out in specific contexts, empowering participants’ to make more informed decisions about their willingness to consent.

Recruitment region was a third important predictor of consent in our sample. Overall 46,5% of participants recruited from the Eastern Cape consented to cell line immortalization in comparison with 37,4% of participants recruited from the Western Cape. This result may speak to the Ubuntu worldview which is particularly prevalent in more traditional Xhosa communities, found most commonly in the rural Eastern Cape. Ubuntu emphasizes the importance of interconnectedness of humans through positive regard for each other and helpful humane interaction. It places emphasises on reciprocity and accountability as key ethical values [18]. The Ubuntu worldview could imply broad support for the concept of sharing of resources, including for instance genomic data and samples in global health research initiatives that may benefit future generations. Participants recruited from the Western Cape where often recruited from more urbanized areas that may be considered less traditional.

While age and region of recruitment were significant predictors of consent, Table 2 suggests that these variables were fairly consistently distributed across recruiters, as was HIV status. Importantly we did not find a significant difference in likelihood to consent between our cases with schizophrenia and unaffected controls, nor did we see a difference between participants based on level of education. Studies suggest that people with schizophrenia generally show poorer understanding of research study elements than healthy controls, despite demonstrating considerable variability in terms of cognitive functioning [18–20], making them potentially more vulnerable to the complexities of consent to cell line storage. However we found that after screening for the quality of understanding of SAX study elements using the UBACC, a range of people recruited as cases with schizophrenia and people recruited as controls with other health-related problems, struggled to demonstrate an adequate understanding of complex SAX study elements after one review of the study consent materials, and benefitted significantly from further explanation of certain elements in the form of iterative learning [14]. This rigorous iterative learning process is likely responsible for why diagnosis and educational level were not predictors of consent in our samples, and could be a valuable resource for managing consent challenges in similar contexts and population groups.

**Table 2:** Recruiter statistics for: age, region recruited from and HIV status

	Age		Region		HIV status	
Recruiter	20–39 years	40–59 years	Eastern Cape	Western Cape	Non-Reactive	Reactive
A (*n* = 308)	193 (63%)	115 (37%)	185 (60%)	123 (40%)	253 (82%)	65 (18%)
B (*n* = 437)	283 (65%)	154 (35%)	243 (56%)	194 (44%)	386 (88%)	51 (12%)
C (*n* = 207)	151 (73%)	56 (27%)	96 (46%)	111 (54%)	181 (87%)	26 (13%)
D (*n* = 130)	103 (79%)	27 (21%)	89 (64%)	41 (36%)	116 (89%)	14 (11%)
E (*n* = 438)	258 (59%)	180 (41%)	265 (60,5%)	173 (39,5%)	399 (91%)	39 (9%)

One important limitation of these results is that we did not measure the time each study recruiter spent on explaining the risks and benefits of cell line creation and immortalization. While study recruiters are provided with a consent script, they typically speak to this material when recruiting participants allowing for a degree of improvisation, and seldom read the script verbatim. The influence of time and attention to specific risks and benefits, particularly in more complex processes like cell lines creation, would be an important consideration when investigating rates of consent. A second limitation is that logistic regression odds ratios do not approximate relative risks for common outcomes. However the same patterns were evident in looking at raw percentages.

## Conclusions

These findings provide guidance on the proportion of participants likely to consent to cell line immortalisation in genomics research, and reinforce the important and influential role that individual recruiters play during the seeking of this consent. Our results also suggest cultural and contextual factors underpinning consent choices, particularly around sharing and reciprocity. Furthermore these results provide support for the growing literature challenging the stigmatizing perception that people with severe mental illness are overly vulnerable as a target group for heath research and specifically genomics studies.

## Additional file

Additional file 1: Database. The original database used for analysis in this manuscript. (XLSX 66 kb)

## Abbreviations

DNA: Deoxyribonucleic acid
HICs: High income countries
LMICs: Low and middle income countries
SAX: ‘Genomics of schizophrenia in South African Xhosa people’ study
SCID-I: Structured clinical interview for DSM-IV axis I disorders
UBACC: University of California, San Diego brief assessment of capacity to consent questionnaire
WHO: World Health Organization
